# Hemoglobin C Disease With Splenomegaly and With Factor II Mutation Gene Thrombophilia: A Case Report

**DOI:** 10.7759/cureus.91937

**Published:** 2025-09-09

**Authors:** Sebastián J Vázquez-Folch, Gabriel A Jiménez-Berríos, Natalio Izquierdo, Victor J Vazquez

**Affiliations:** 1 School of Medicine, Universidad Central del Caribe, Bayamón, PRI; 2 School of Medicine, Universidad Central del Caribe, Bayamon, PRI; 3 Department of Surgery, School of Medicine, Medical Sciences Campus, University of Puerto Rico, San Juan, PRI; 4 Comprehensive Cancer Center, Medical Sciences Campus, University of Puerto Rico, San Juan, USA

**Keywords:** factor ii mutation, genetic thrombophilia, hemoglobin c, hemolytic anemia, splenomegaly

## Abstract

Hemoglobin C (Hb C) disease is caused by a mutation that leads to reduced hemoglobin solubility, crystallization, and chronic hemolysis. We report on a patient with a homozygous Hb C disease, persistent fetal hemoglobin, and a heterozygous factor II mutation. This case illustrates the complex interaction between hemoglobinopathies and thrombophilia disorders, where the coexistence of Hb C disease and a factor II mutation significantly heightens the risk of thrombosis. Its geographic prevalence in the Caribbean, particularly Puerto Rico, reflects historical migration patterns and underscores the need for early detection and multidisciplinary care.

## Introduction

Hemoglobin C (Hb C) disease is an autosomal recessive hemoglobinopathy caused by a mutation in the beta-globin (*HBB*) gene, in which glutamic acid is substituted with lysine at position 6 of the beta-globin chain [1]. This mutation leads to reduced hemoglobin solubility, hemoglobin crystallization within red blood cells (RBCs), and chronic hemolysis [2]. The disease primarily affects individuals of African, Caribbean, and Mediterranean descent, with Hb C trait (heterozygous state) being relatively common, but homozygous Hb C disease (Hb CC) being less frequent [3].

Patients with homozygous Hb C disease may have mild to moderate hemolytic anemia, accompanied by splenomegaly, the presence of target cells on peripheral blood smears, and occasional Hb C crystal formation within RBCs [4]. While the disease course is generally mild compared to other hemoglobinopathies such as sickle cell disease (SCD), complications such as gallstones, splenic sequestration, and rare thrombotic events may occur [5].

An additional factor of clinical importance in hemoglobinopathies is hereditary thrombophilia. The prothrombin G20210A (factor II) mutation is a well-documented genetic variant associated with an increased risk of venous thromboembolism (VTE) [6]. Patients with this mutation, particularly in the heterozygous state, exhibit elevated plasma prothrombin levels, predisposing them to thrombotic complications [7]. The coexistence of Hb C disease with a thrombophilic disorder, such as factor II mutation, raises concerns regarding increased thrombotic risk, and careful clinical management is warranted [8].

We report on a patient with homozygous Hb C disease, persistent fetal hemoglobin (HPFH), and a heterozygous factor II mutation, highlighting the potential interplay between hemoglobinopathies and thrombophilia. By examining this case in detail, we aim to contribute to the understanding of the clinical implications, management strategies, and potential complications arising from the coexistence of these genetic hematologic diseases. In addition, we explore the genetic basis of Hb C disease and factor II mutation and their individual and combined effects on hematological parameters and thrombotic risk.

## Case presentation

Patient information

A 45-year-old female patient was referred for evaluation due to splenomegaly and abnormal hemoglobin electrophoresis findings. The patient had a medical history notable for Hb C disease, persistent HPFH, and factor II thrombophilia. She had a history of one miscarriage (G2P1A1).

History of the present illness

The patient initially had occasional bone pain and abdominal discomfort. Routine laboratory evaluations and genetic testing are shown in Table 1 and Table 2.

**Table 1:** Patient's laboratory and hemoglobin electrophoresis results Hb: hemoglobin, MCV: mean corpuscular volume, LDH: lactate dehydrogenase, PT: prothrombin time, PTT: partial thromboplastin time, µL: micro liters

#	Test	Result	Reference values
1	Hb C	88.2% (repeat: 87.7%)	0% (absent in healthy individuals)
2	Hb A2	2.3% (normal)	2.0–3.5%
3	Hb F	9.5% (persistent fetal hemoglobin)	<1% (adults); up to 2% in some labs
4	Platelet count	200,000 platelets/µL	150,000–450,000 /µL
5	MCV	77 fL	80–100 fL
6	Reticulocyte count	3%	0.5–2.5% (or 25,000–100,000 /µL absolute)
7	LDH	147 U/L	140–280 U/L
8	Prothrombin time (PT)	10.9 sec	11–13.5 seconds
9	INR	1	0.8–1.2 (for patients not on anticoagulants)
10	PTT	27.5 sec	25–35 seconds

**Table 2:** Genetic testing Hb C: hemoglobin C

#	Test	Result
1	Homozygous Hb C disease	Homozygous for a pathogenic variant (c.19G>A,p.E7K) of the *HBB* gene
2	Heterozygous factor II mutation (G20210A)	Increased risk for thrombophilia
3	Alpha-thalassemia (HBA1/HBA2) testing	Negative

**Figure 1:**
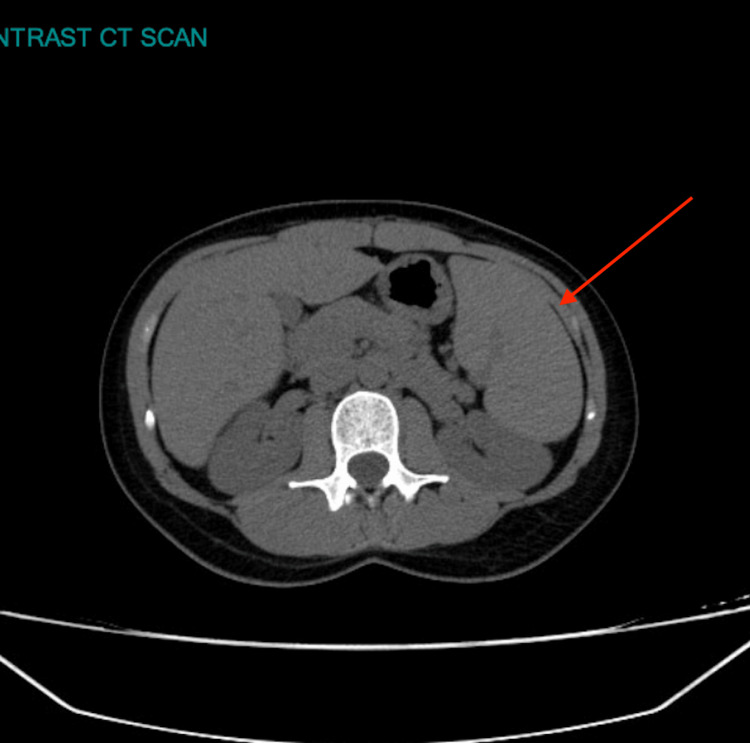
Axial CT scan of the abdomen The red arrow is pointing at the splenomegaly.

**Figure 2:**
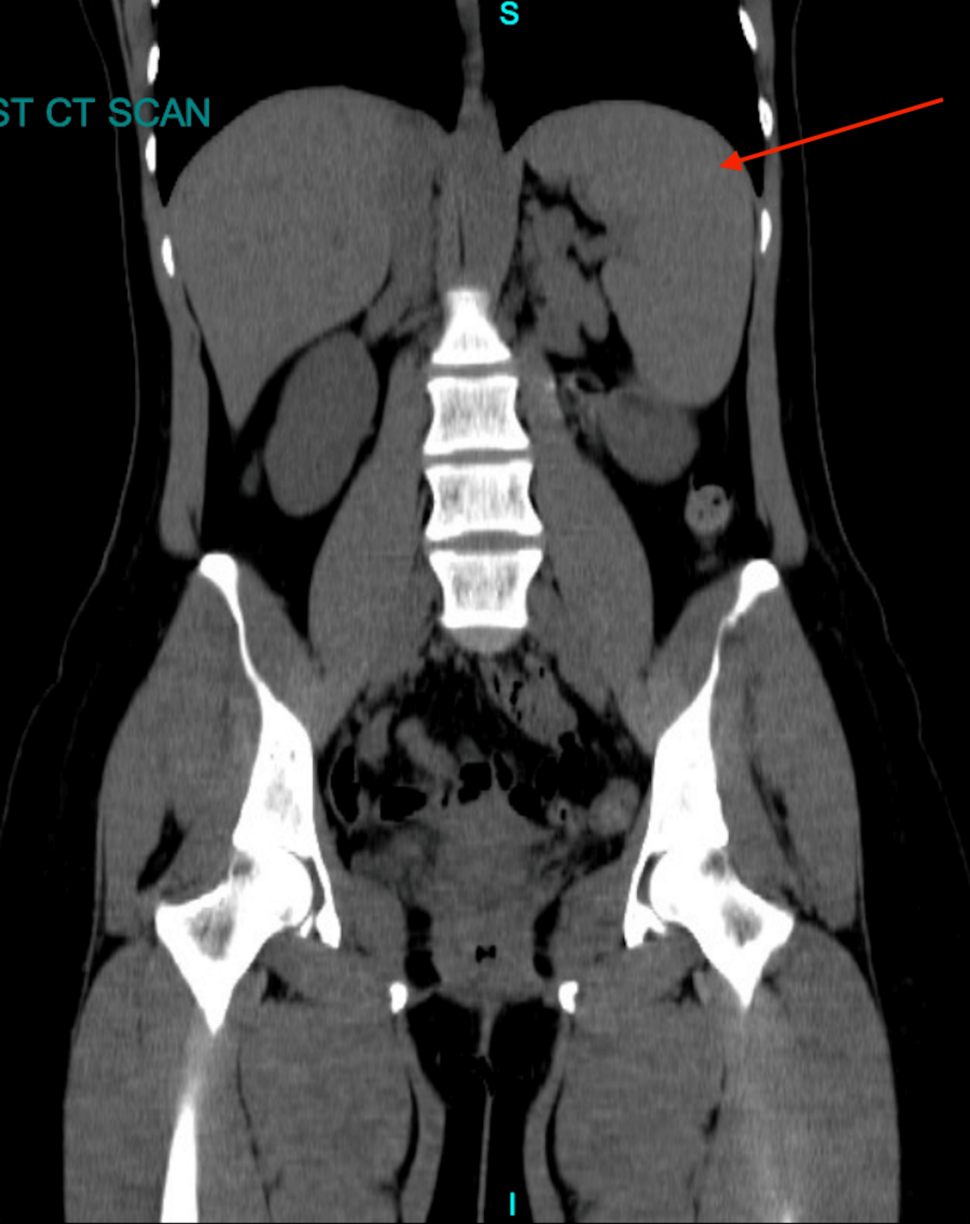
Coronal view of the abdomen CT scan The red arrow is pointing at the spleen, which shows splenomegaly.

Splenomegaly with a size of 18 cm was seen on the CT scan of the abdomen and pelvis. Axial and coronal views are shown in Figures 1-2.

Clinical assessment and management plan

Given the coexistence of Hb C disease and factor II mutation, the patient was determined to be at increased risk for venous thromboembolism (VTE). Despite normal coagulation parameters, thrombosis risk remains a long-term concern. 

The patient was recommended to continue daily folic acid supplementation, encouraged to increase hydration, and oriented to consider genetic counseling to discuss reproductive risks and thrombophilia management. An ophthalmology referral to evaluate for Hb C-associated retinopathy was given. The patient was closely followed up to monitor splenomegaly progression and potential thrombosis.

The patient was reassured that the Hb C disease is generally not a critical illness but requires lifelong monitoring due to splenomegaly and thrombophilia risk. The importance of thrombosis prevention measures and possible future anticoagulation therapy were discussed.

## Discussion

Hb C is a hemoglobin variant that is presumed to have originated in West Africa and Southeast Asia, with its highest prevalence in regions such as Ghana and Burkina Faso [9]. The presence of the Hb C trait in these regions reaches up to 25%, making it one of the most common hemoglobinopathies in these populations [9]. The high prevalence of Hb C in these regions suggests a potential evolutionary advantage, similar to hemoglobin S (Hb S) in sickle cell disease [10].

Migration of African populations during the transatlantic slave trade probably contributed to the introduction of Hb C into the Caribbean, South America, and the United States [9]. In the Caribbean, particularly in Puerto Rico, Hb C has been reported in individuals of African ancestry. It occurs with a prevalence of approximately 3.5% among the Afro-Caribbean population [9]. The genetic admixture in Puerto Rico, which includes African, European, and Indigenous Taíno ancestry, has influenced the distribution of hemoglobinopathies, including Hb C and Hb S, in the region [3].

Patients with homozygous Hb C disease (Hb CC) typically have mild to moderate hemolytic anemia, with symptoms including splenomegaly, Hb C crystals in red blood cells, and increased target cells on peripheral blood smears [2]. Compared to sickle cell disease, Hb C disease has a less severe clinical presentation, although it may still contribute to chronic hemolysis and gallstone formation [5].

The coexistence of Hb C disease with factor II thrombophilia, as seen in this patient, is clinically significant. The prothrombin G20210A mutation is a known prothrombotic condition, increasing the risk of venous thromboembolism (VTE), including deep vein thrombosis (DVT) and pulmonary embolism [6]. While Hb C disease alone does not typically predispose patients to thrombotic events, the additional presence of factor II mutation warrants careful clinical monitoring [7]. However, the patient’s persistent HPFH may provide a protective effect against severe hemolysis, as increased Hb F levels are known to modulate disease severity in hemoglobinopathies like sickle cell disease [10]. However, the relationship between HPFH and thrombotic risk remains unclear.

The management of Hb C disease with coexisting thrombophilia requires a multidisciplinary approach, involving hematologists, geneticists, and peripheral vascular surgeons. Close surveillance for thrombotic events is essential, as patients with factor II mutation are at an increased risk of VTE, particularly in situations such as immobility, pregnancy, or surgery [6]. 

The patient's history of one miscarriage may be associated with her underlying thrombophilia. Studies have demonstrated that carriers of the prothrombin G20210A mutation have an increased risk of recurrent pregnancy loss (RPL). This association underscores the importance of genetic counseling and tailored management strategies during pregnancy to mitigate risks [11]. Even in the absence of overt coagulation abnormalities, preventive strategies and close monitoring are necessary to mitigate potential thrombotic complications.

Another critical aspect of management is monitoring and addressing splenomegaly, a common feature of patients with Hb C disease [3]. In this case, the patient's massive splenomegaly poses a potential risk for splenic sequestration and hypersplenism, which can lead to complications such as anemia, thrombocytopenia, and increased hemolysis [2]. Regular clinical evaluation and imaging are necessary to assess spleen size and function, and in severe cases, splenectomy may be considered.

Genetic counseling plays a pivotal role in the management of patients with Hb C disease and factor II mutation. Given the hereditary nature of both diseases, genetic counseling is crucial for risk management and reproductive planning. This is particularly important in family planning discussions, as prospective parents with Hb C and thrombophilic mutations must be informed of the potential genetic implications for their offspring [10,11].

Another important consideration is the potential for Hb C-associated retinopathy [12]. Ophthalmologic evaluation is recommended as part of routine care, given the association of Hb C disease with retinal changes. Regular eye exams help monitor for early signs of retinopathy, allowing for timely intervention to prevent visual complications [13]. In this particular case, the patient has already undergone an ophthalmologic evaluation and had no retinal manifestations of the disease.

## Conclusions

This case highlights the complex interplay between hemoglobinopathies and thrombophilic disorders, emphasizing the need for individualized co-management strategies. While Hb C disease is often considered benign, the concurrence of factor II mutation significantly increases the patient’s risk for thrombotic complications. 

The geographic distribution of Hb C in the Caribbean, particularly in Puerto Rico, reflects historical population migrations and genetic admixture. Early identification and multidisciplinary management are essential in optimizing outcomes for patients with Hb C disease.
